# Integration of machine learning models with microsatellite markers: New avenue in world grapevine germplasm characterization

**DOI:** 10.1016/j.bbrep.2024.101678

**Published:** 2024-03-10

**Authors:** Hossein Abbasi Holasou, Bahman Panahi, Ali Shahi, Yousef Nami

**Affiliations:** a Department of Plant Breeding and Biotechnology, Faculty of Agriculture, University of Tabriz, Tabriz, Iran; b Department of Genomics, Branch for Northwest and West Region, Agricultural Biotechnology Research Institute of Iran (ABRII), Agricultural Research, Education and Extension Organization (AREEO), Tabriz, Iran; c Faculty of Agriculture (Meshgin Shahr Campus), Mohaghegh Ardabili University, Ardabil, Iran; d Department of Food Biotechnology, Branch for Northwest and West Region, Agricultural Biotechnology Research Institute of Iran (ABRII), Agricultural Research, Education and Extension Organization (AREEO), Tabriz, Iran

**Keywords:** Feature selection, Machine learning, Microsatellites, Vitis

## Abstract

Development of efficient analytical techniques is required for effective interpretation of biological data to take novel hypotheses and finding the critical predictive patterns. Machine Learning algorithms provide a novel opportunity for development of low-cost and practical solutions in biology. In this study, we proposed a new integrated analytical approach using supervised machine learning algorithms and microsatellites data of worldwide vitis populations. A total of 1378 wild (*V. vinifera* spp. *sylvestris*) and cultivated (*V. vinifera* spp. *sativa*) accessions of grapevine were investigated using 20 microsatellite markers. Data cleaning, feature selection, and supervised machine learning classification models vis, Naive Bayes, Support Vector Machine (SVM) and Tree Induction methods were implied to find most indicative and diagnostic alleles to represent wild/cultivated and originated geography of each population. Our combined approaches showed microsatellite markers with the highest differentiating capacity and proved efficiency for our pipeline of classification and prediction of vitis accessions. Moreover, our study proposed the best combination of markers for better distinguishing of populations, which can be exploited in future germplasm conservation and breeding programs.

## Highlights

• VVMD32_1_243, VVIn73_1_257, VVIp60_1_302, and VVMD7_1_235 alleles were the most important features.
• SVM were even more successful algorithms in predicting and categorizing accessions among the two and nine expected populations.
• Presence of VVMD32-271, −259 and −257 alleles separate wild and cultivated accessions of grapevines.

## 1 Introduction

Over the last decade, advances in molecular biology technologies have led to tremendous growth in biological data. Among biology technologies, a wide range of molecular techniques has been developed for genetic diversity and germplasm characterization of organisms [[1], [2], [3], [4], [5]]. These data present the raw material needed to gain insights into the hidden layer of molecular diversity data. However, the potential of these data can only be realized through next-level analyses [6]. On top of that, the development of new analytical models for interpretation and understanding of these biological processes to take new perspectives, generate novel hypotheses, and find critical predictive patterns. Among different modeling approaches, Machine Learning algorithms provide numerous opportunities for development of low-cost and practical solutions [[7], [8], [9]]. Machine learning is an area of artificial intelligence that is integrated with statistical and computational methods to automatically learn from data. The learning process itself refers to knowledge discovery that translate the features in the training data into pattern, and clustering/prediction of the labels [10,11].

Machine learning is divided into two overarching categories *viz*., supervised and unsupervised learning methods [12]. Unsupervised machine learning methods are used when the labels on the input data are unknown; these methods learn only from patterns in the features of the input data. In supervised methods, on the other hand, labeled features are trained to predict the class labels based on training examples. Among a large number of supervised models reported, decision trees, naive Bayes, and support vector machines (SVMs) are simple and effective methods with a broad range of application in biology [8,9,[12], [13], [14], [15]].

SVM is the most popular supervised learning algorithms, which uses kernel function to project data into a higher dimensional space to classify data. In other words, SVM is based on the concept of decision planes that define decision boundaries between different class members [12,15]. Decision trees are predictive models that are performed under uncertain conditions in a recursive manner. Decision trees are made of a root, internal, or non-leaf node (test on attributes) and leaf nodes (label class) [12,14]. The Naive Bayesian classifier is expanded based on Bayes’ theorem with features independence hypothesis. Despite easy to implement, Naive-Bayes classifier is known as highly sophisticated classifiers [7,16].

Grapevine has had a noble gift of nature to the mankind and cultural importance for the Iranians through millennia. Grapevine, as the most widely grown fruit plants in the world, is recognized as the earliest domesticated fruit plants in the world nowadays [[17], [18], [19], [20], [21]]. *Vitis*, is the commonly cultivated grapevine in the worldwide, ranges from Central Asia to the Mediterranean Basin [21]. Within the genus Vitis, *V. vinifera* is the primary species used in the viticulture for the large-scale production of table fruits, raisins, juice, and wine [18]. Two subspecies sylvestris and sativa have been described for *V. vinifera*, which includes the wild populations and cultivated/domesticated varieties, respectively [22]. Grape domestication occurred in the upland regions of Eastern Turkey and in the northwest of Iran about 6000–8000 years ago [23,24]. From there that domesticated grapevines spread to Southern Balkans and East Mediterranean Basin. During the first millennium, BCE grapevine appeared in Sicily, Western and Central Europe. Then, grapevine cultivation reached Central and South East Asia (This et al., 2006; [22]). Despite the many studies of genetic diversity and research on grapevine domestication history and its spread, but this proposition has remained mysterious, until now. Recently, a study with molecular mechanism in 3525 cultivated and wild accessions suggested that grapevine domestication occurred concurrently about 11,000 years ago in Western Asia and the Caucasus to yield table and wine grapevines [21].

The cultivated grape *V. vinifera* subsp. *sativa* has had a great economic impact all over the world. However, because of human population growth, destruction of habitats, and natural phenomena such as floods, fire and pathogen dispersal, the wild grape *V. vinifera* subsp. *sylvestris*, is in danger of extinction currently. Hence, there is urgent need to characterize and conserve grape germplasm for future programs. So far various molecular markers, such as SSR [22,[25], [26], [27], [28], [29], [30], [31], [32], [33], [34], [35], [36]], SNP [20,22,28,[37], [38], [39], [40], [41]], AFLP [42], Retrotransposon [43,44] and ISSR [31] have been used to characterize different grapevine accessions. However, because of considerable genetic diversity and synonyms (variety of names for the same genotype) or homonyms (same name for different genotypes) in the clonal propagated grapevines, characterizations of the accessions are still challenge. Although molecular markers especially SSR and SNP are effective methods to characterization and classifying the worldwide grapevine germplasm. Nevertheless, machine learning (ML) approaches, which efficiently facilitate pattern recognition and classification leading to prediction by creating models using existing data. Therefore the integration of molecular markers with machine learning approaches could help to classification and prediction by creating models using existing data of grapevine for future diversity and conservation programs.

The data produced in Riaz et al. [30] provides valuable information of microsatellites profiles for Caucasus, Central Asia, and the Mediterranean basin vitis collections. In order to determine the most indicative markers for distinguishing among diverse vitis populations and subspecies, we assessed machine learning based modeling approach on these data sets. The main objective of this study was to evaluate feasibility and efficiency of supervised machine learning algorithms in classification and prediction of worldwide vitis populations based on microsatellites data sets. We show that the integrated pipeline used in this study is highly reliable in classifying and predicting world grapevine accessions.

## 2 Materials and methods

### 2.1 Datasets

A total of 1378 wild (*V. vinifera* spp. *sylvestris*) and cultivated (*V. vinifera* spp. *sativa*) accessions of grapevine across different regions of central Mediterranean and Central basin were subjected to 20 microsatellite markers (namely; VMC1b11, VMC4f3.1, VVIb01, VVIh54, VVIn16, VVIn73, VVIp31, VVIp60, VVIq52, VVIv37, VVIv67, VVMD21, VVMD24, VVMD25, VVMD27, VVMD28, VVMD32, VVMD5, VVMD7, VVS2) analysis [30]. The datasets belonged to nine countries including Turkmenistan, Pakistan, Georgia, Armenia, Azerbaijan, Croatia, Spain, France and Italy. Table 1 provides the details of accessions that were included in this study.

**Table 1:** Details regarding the 1378 accessions of grapevine used in this study from the different geographical regions of the world.

Country	Accessions	
	*V. vinifera* spp. *sylvestris*)	*V. vinifera* spp. *sativa*
Spain	192	145
Italy	289	34
France	46	32
Georgia	76	112
Turkmenistan	–	59
Pakistan	–	14
Croatia	38	–
Armenia	49	–
Azerbaijan	292	–
Total	982	396

### 2.2 Data processing

In data cleaning step, at first, allelic profiles for all accessions were converted into yes/no binomial variables, assigning ‘yes’ for the present allele and ‘no’ for all other absent alleles at each locus. Next, correlated (correlation coefficient higher than 0.95), and useless attributes (above and below percent of examples) were removed from initial data sets. Hereafter the processed data sets were called Pdb (Processed database). The Pdb were then subjected to additional analysis. In this study, two different experiments for computational analyses were designed and carried out. In the first experiment, here called the 2-targeted (2-t) experiment, subspecies were used to divide datasets into wild and cultivated categories. Second experiments, here called the 9-targeted (9-t) experiment, were designed to assess the differentiation power of the informative loci to assign each population to the geographical origin. In the 9-t experiment, nine different countries were defined as nine different geographically targets for analyses.

### 2.3 Features selection with weighting algorithms

The main objective of feature selection is to select a subset of most informative and non-redundant features that can increase the modeling performance [45]. For selection of the most indicative and informative features (alleles), seven weighting algorithms, including Super Vector Machine (SVM), Chi-Square, Gini Index, Information Gain Ratio, Information Gain, Uncertainty and PCA were implied on the Pdb. Attribute weighting results were normalized between 0 and 1 and the attributes with values higher than 0.5 were considered as indicative attribute. Results of weighting algorithms were used for creation of distinct data set.

### 2.4 Prediction and classification with supervised ML methods

**Fig. 1:**
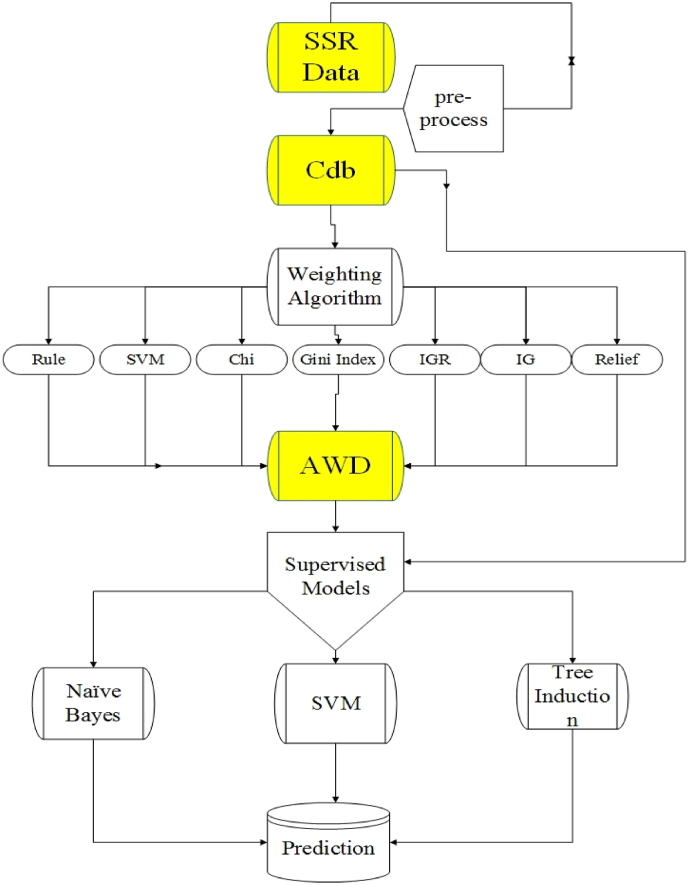
Flowchart of the data analysis, which shows the structure of the analytical approach to the investigation of microsatellite (SSR) markers in this study.

Seven data sets of attribute weighting steps plus the Pdb were separately implied for prediction and classification with three supervised methods, including the Naive Bayes, SVM and Tree Induction. In order to construct the most accurate decision trees, four decision tree algorithms *viz*., Decision Tree, Decision Stump, Random Tree, and Random Forest with four different criteria (Gain Ratio, Information Gain, Gini Index and Accuracy) were separately run on each eight databases, and the mean of accuracy was reported. In the Naive Bayes algorithm, two models namely Naive Bayes (returns classification model using estimated normal distributions) and Naive Bayes kernel (returns classification model using estimated kernel densities) with four Gain Ratio, Information Gain, Gini Index and Accuracy criteria were run. Regarding the SVM algorithm, four kernels, including the ref, sigmoid, linear, and poly were tested on data sets in two experiments. To avoid over fitting of models, performance of the models was evaluated with 10-fold cross validation. In both experiments, 90% of the data were set as training and remaining 10% were used as test data. This procedure was repeated 10 times (10-folds) and the accuracy of prediction and classification was defined by taking the percentage of correct predictions over the total number of examples. Workflow of the implemented pipeline was presented in Fig. 1.

## 3 Results

### 3.1 Allele identification and allele frequency determination

Alleles’ frequency was screened across 20 microsatellite loci. Among 412 scored alleles, VMC4f3 and VVMD28 with 31 and VVIq52 with 11 alleles were detected as the most and least variable loci, respectively (Table 2).

**Table 2:** Microsatellite allele lengths, loci and the total alleles.

Locus	Allele lengths (bp)	Total alleles
VVIp60	320-289-316-298-328-318-302-310-314-306-272-300-312-322-304-324-279-330-326-308	20
VVMD28	235-243-233-245-217-257-277-247-271-227-275-239-225-253-263-215-255-251-259-267-261-265-231-223-241-249-237-219-280-269-273	31
VVIb01	290-294-288-298-272-316-278-284-308-292-286-302-300-296-304-306-312-318-324-310	20
VVMD27	179-187-185-195-181-175-183-189-193-191-177-213-207-171-217-211-203-201-197-219	20
VVIv67	354-356-348-360-358-336-368-344-350-364-352-374-346-372-324-362-370-332-334-366-342-255-376-386-378-384	26
VVMD32	257-271-251-243-239-247-255-249-261-265-245-253-241-263-259-267-273-269-277	19
VVIn16	149-151-147-157-141-155-145-159-153-172-168-161-156	13
VVMD21	241-255-247-249-226-219-245-253-251-230-239-243-237-257-265-263-271-261	18
VVIv37	159-149-167-155-153-145-173-147-157-163-151-141-177-161-165-179-143-169-175-171-176	21
VVMD24	206-210-214-216-208-204-212-202-196-194-200-218	12
VVMD7	235-247-243-249-233-251-239-253-259-241-245-263-237-267-257-261-231-265-255-269	20
VMC1b11	165-181-173-169-183-187-193-185-171-167-175-197-177-163-191-157-155-151-199-195-179-189	22
VVS2	133-125-143-139-137-135-141-151-131-145-149-123-147-153-157-159-155-129-167-161	20
VVMD5	240-228-234-238-232-226-236-230-224-265-252-222-242-244-248-246-250-267-263-233	20
VVIn73	257-263-267-265-259-253-261-255-271-251-273-270-275-269	14
VVIp31	184-166-172-186-188-190-192-174-182-178-170-180-176-196-204-200-194-213-202-164-158-161-198-215-217-206	26
VVIh54	147-151-139-165-167-159-175-163-153-129-155-149-145-143-157-161-137-131-169-141-173-171-179-177-181	25
VVIq52	80-78-84-76-82-88-86-74-66-72-68	11
VMC4f3.1	172-164-182-186-188-178-166-158-204-174-176-170-206-202-180-208-149-194-168-196-153-156-143-210-190-184-192-212-200-233-179	31
VVMD25	238-254-240-248-242-244-266-250-262-236-252-256-270-260-246-272-239-268-258-264-275-261-257	23
Total		412

### 3.2 Data cleaning

Among the investigated SSRs, 17 loci with above 50% effective alleles higher were included for further analyses. These alleles included VMC1b11, VMC4f3, VVIb01, VVIh54, VVIn16, VVIp31, VVIp60, VVIq52, VVIv37, VVMD21, VVMD24, VVMD27, VVMD28, VVMD32, VVMD5, VVMD7 and VVS2.

### 3.3 Feature selection by weighting algorithms

Seven attributes weighting algorithms (AWA) were applied on Pdb and gave feature weight values between 0 and 1. The weight value higher than 0.5 % was implied as selective criteria in both experiments. In the 2-t experiment, VVMD32-271 was the most important allele pointed out by 6 AWAs, followed by VVMD7-263, VVSO2-147, VVMD27-179, VVMD21-253, VVIq52-78, VVMD27-189, VVIh54-165, VVMD5-232 and VVMD28-243. Weighted values for all alleles were presented in supplementary Table S1. In the 9-t experiment, VVIh54_1_139, VVMD21_1_249, VVMD21_2_249, VVMD32_1_247, and VVMD32_2_247 were the most important alleles pointed out by all AWAs. Moreover, importance of VVMD32_1_243, VVIn73_1_257, VVIp60_1_302, and VVMD7_1_235 alleles were confirmed by more than three AWAs (supplementary Table S1).

### 3.4 Machine learning prediction of target populations

#### 3.4.1 Tree induction models

The performances among 416 tree induction models *viz*, Decision Stump, Decision Tree, Decision Parallel and Random Forest Tree, with 4 different criteria including the Gain ratio, Information gain, Gini index and Accuracy run on eight different data sets ranged from 24 to 86 % for both experiments (Table 3). In the 2-t experiment, the highest (86.87%) and lowest (71.26 %) performance gained when Decision tree run with Information Gain and Decision Stumps run with Gini index respectively (Table 3). Prediction rates aforementioned algorithms in the 2-t experiment are presented in Table 4, where 304 *Sativa* accessions out of 396 and 893 *Sylvestris* accessions out of 982 were correctly predicted. However, 92 *Sylvestris* accessions were predicted as *Sativa* accessions.

**Table 3:** The performance of induction tree models on Pdb computed at 10-fold cross validation for both experiments.

Models	2-t experiment				9-t experiment			
	Gain Ratio	Information Gain	Gini Index	Accuracy	Gain Ratio	Information Gain	Gini Index	Accuracy
Decision Tree	85.92	86.87	85.56	85.34	50.87	63.43	57.4	71.84
Decision Stump	80.48	80.48	71.26	71.26	25.11	39.48	24.46	24.46
Random Forest	71.26	71.26	71.26	71.26	47.1	27.79	28.81	39.04
Random Tree	71.70	71.70	73.22	71.70	24.46	24.46	31.93	31.28

**Table 4:** Prediction rate (accuracy) details of decision tree (using information gain criteria) with 10-fold cross validation for each types in the 2-targeted (2-t) experiment.

True Predicted	*V. vinifera subps. Sativa*	*V. vinifera subps. Sylvestris*
*V. vinifera subps. Sativa*	304 (out of 396)	89
*V. vinifera subps. Sylvestris*	92	893 (out of 982)

**Fig. 2:**
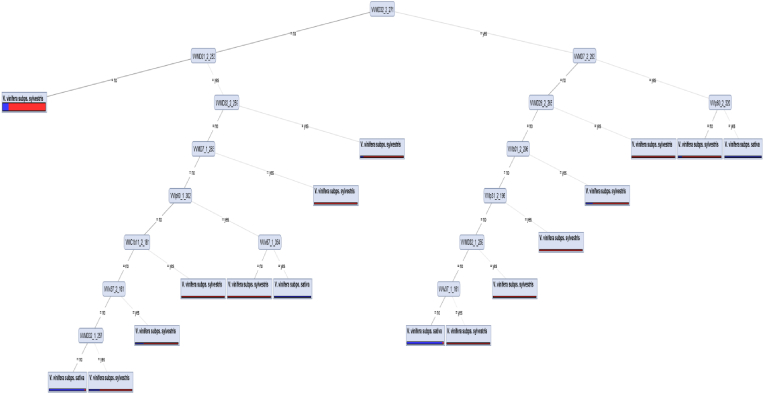
Decision Tree generated model showing separation of wild and cultivated grape populations in the 2-targeted (2-t) experiment.

Fig. 2 illustrates the tree constructed by the Decision Tree model based on Pdb for the 2-t experiment. VVMD32-271 was the root feature and the most important feature. As shown in Fig. 2, presence of any of the VVMD32-271, −259 and −257 alleles would help to separate wild and cultivated accessions of grapevines. Absence of VVMD32-271, −259 and −257 alleles and presence of VVMD28-265, VVMD32-259, VVMD7-263, VVMC1b11-181, VVIv37-161, VVIb01-296, or VVIp31-196 would be categorized the grapevines as cultivated (*Sativa*) subspecies.

In the 9-t experiment, the highest (86.87%) and lowest (71.24 %) performance gained when Decision Tree run with accuracy criteria and Random Tree run with Information Gain, respectively (Table 3). Predicted details for Decision Tree run with accuracy criteria are presented in Table 5, where 75 out of 188 accessions from Georgia, 25 out of 49 accessions from Armenia, 262 out of 292 accessions from Azerbaijan, 335 out of 337 accessions from Spain, and 170 out of 323 accessions from Italy were predicted correctly (Table 5). Croatia samples were all correctly predicted.

**Table 5:** Prediction rate (accuracy) details of each decision tree with 10-fold cross validation for each of the types in the 9-targeted (9-t) experiment.

True Predicted	Turkmenistan	Pakistan	Georgia	Armenia	Azerbaijan	Croatia	Spain	France	Italy
Turkmenistan	39	1	2	2	1	0	1	0	0
Pakistan	0	7	2	1	0	0	0	0	0
Georgia	7	1	175	1	5	0	1	0	0
Armenia	3	0	2	30	4	0	0	0	2
Azerbaijan	4	2	1	2	262	0	0	0	3
Croatia	0	0	0	0	0	38	0	0	0
Spain	5	2	6	9	15	0	335	0	48
France	0	0	0	0	0	0	0	77	0
Italy	1	1	0	4	5	0	0	1	270

**Fig. 3:**
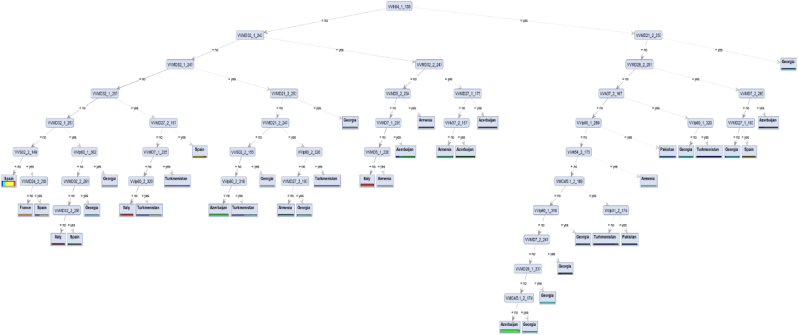
Decision Tree generated model showing separation of grape populations in the 9-targeted (9-t) experiment.

As shown in Fig. 3, in the 9-t experiment VVh54-139 allele was defined as root feature for the constructed decision tree. In combination with VVMD21-253 allele, the tree was able to classify accessions from Georgia, while absence of allele VVMD28-257 combined with the presence of allele VVMD7-263 identified accessions from Azerbaijan country.

#### 3.4.2 Support vector machine (SVM) approach

In this study, SVM was used with RBF, Sigmoid, Linear and Poly as the kernel function. In the 2-t experiment, highest and lowest overall accuracy of different SVM models ran with different kernel types were in the range of 71.26–97.46 % for the 2-t experiments and 24.46–92.53% for the 9-t experiment (Table 6).

**Table 6:** The total accuracy obtained from running SVM (C-SVC) method.

	kernel type	Radial Basis Functions (RBF)	Sigmoid	Linear	Polynomial
2-t experiment		97.46	71.26	95.07	96.03
9t-experiment		92.53%	24.46%	78.81%	88.82%

#### 3.4.3 Naive Bayes

The accuracies of Naive Bayes and Naive Bayes Kernel models ran on seven datasets for two designed experiments were presented in Table 7. In the 2-t experiment, the lowest accuracy (84.03%) gained when both Bayesian models ran on PCA dataset, whereas the best accuracy (96.81%) gained when Naive Bayes and Naive Bayes Kernel models ran on Pdb. In the 9-t experiment, the lowest accuracy (31.20%) gained when Naive Bayes kernel model ran on SVM dataset. However, the best accuracy (93.69%) gained when Naive Bayes and Naive Bayes kernel models ran on Pdb.

**Table 7:** The accuracy of Bayesian model on various datasets computed by 10-fold cross validation.

Dataset	9-t experiment		2-t experiment	
	Naive Bayes Kernel	Naive Bayes	Naive Bayes Kernel	Naive Bayes
Pdb	93.69%	93.69%	96.81%	96.81%
Info Gain Ratio	58.64%	58.64%	90.78%	90.78%
Info Gain	67.49%	67.49%	86.79%	86.79%
SVM	31.20%	71.99%	94.63%	91.8%
Gini	65.46%	65.46%	88.24%	88.24%
PCA	90.28%	87.45%	84.03%	84.03%
Chi Squared	63.86%	63.86%	84.54%	84.54%

## 4 Discussion

The predictive ability and robustness of ML algorithms has proven superior to statistical and classical methods such as principal component analysis (PCA) and cluster analysis in many studies [46]. In particular, ML algorithms have been successfully applied to find specific molecular markers for prediction of olive [47,48], wheat [49] cultivars. Due to their reduced application time, high predictive performance and generalization capabilities, ML algorithms are becoming a valuable tool for data mining.

In this study, five loci namely VVMD7, VVMD32, VVMD21, VVS2, and VVIq52 from a starting set of 20 loci were selected based on their efficiency in characterizing the two subspecies, as defined by the entire attribute weighting algorithms. The informative features of VVS2, VVMD7, VVMD32, VVMD5 and VVIq52 have been reported by previous studies [25,26,31,50,51].

Doulati-Baneh et al. [26] have demonstrated that VVS2 and VVMD7 loci are able to differentiate 67 Iranian cultivars and landraces. Wang et al. [27] reported that VVMD7 and VVMD32 are the most indicative loci among 49 accessions of grape genotypes originating from different countries. De Andres et al. [25] also reported that VVS2 and VVMD7 are the most indicative locus among 237 Spanish cultivars.

Genetic diversity of grapevine has been characterized using different molecular markers through several studies [[25], [26], [27],^31^,^43^,^50^]. However, finding ranked patterns/combinations of molecular markers that may provide higher efficiencies for differentiating among grapevine accessions has not been attempted up to now. Supervised machine learning models are methods of choice for this purpose. This is the first study, to the best of our knowledge, which is reporting application of ML models to find the best indicative and informative combination of candidate SSR markers in world grapevine accessions. Our findings has distinguished world wild and cultivated grapevine accessions via introducing the most indicative distinguishing alleles. Diago et al. [52] and Fernandes et al. [53] utilized hyper spectral imaging for the varietal classification of grapevine leaves and clones respectively.

As shown in Table 3, the overall accuracies for tree induction models were generally high for all algorithms. Precision of wild accessions prediction is more than cultivated accessions prediction except when the Decision Tree model ran with Gain Ratio and Decision Stump model ran with Gain Ratio and Information Gain.

With an increase in the number of target groups from the first (2-t) to the second (9-t) experiment, an increase in the number of informative loci was observed. According to our finding, VVIh54-139 and VVMD32-271 that are located at the top of the tree hierarchies (Fig. 2, Fig. 3) have adequate abilities to separate and shape the topology; furthermore, construct patterns of the marker-based discrimination. In this respect, Beiki et al. [47] analyses showed that ISSR loci UBC841a4 were the superior attributes in making classification among foreign and domestic olive cultivars with 100% accuracy. Torkzaban et al. [48] have shown that DCA14-149, DCA9-206 and DCA16-178-2 have enough potential to make an obvious discriminative pattern between different olive accessions.

Bayesian algorithms were even more successful than the decision trees in predicting and categorizing accessions within the two and nine expected populations. Naive Bayes and Naive Bayes Kernel retrieved an accuracy of 90.98% and 96.81% for 9-t and 2-t, respectively (Table 7). Riaz et al. [30] reported that the Bayesian analysis of the population structure did not have a clear separation between wild (*sylvestris*) and cultivated grapevines (*sativa*). While previous studies gave a polymorphism pattern across the world grapevine populations, the present study has provided details on this diversity by assessing the effectiveness of the polymorphic loci in the characterization of those populations by employing useful machine learning methods. Although both Bayesian models (Naive base and Naive base kernel) have shown similar accuracies in predicting the grapevine accessions, the Naive Bayes Kernel model appears to perform better when it is applied to the SVM dataset in 2-t experiment, and PCA dataset in 9-t experiment (Table 7).

SVM were even more successful than the Tree Induction and Naive Bayes algorithms in predicting and categorizing accessions among the two and nine expected populations for the 2-t and 9-t experiments.

## 5 Conclusion

To put it to sum up, various supervised algorithms were applied in this research to uncover the most suitable computational and analytical tools to identify groups of alleles with similar patterns in making precise discrimination among wild/cultivated and world grapevine accession based on SSR data. This study displayed that the SSR loci VVIh54-139 and VVMD32-271 were more indicative attributes in classification among different subspecies of grapevine. This study for the first time shows that allele feature in combination with machine learning algorithms can effectively classify grapevine accessions of geographically separated accession of grapevines based on SSR profiles.

## Funding information

No funding was received for current study.

## CRediT authorship contribution statement

**Hossein Abbasi Holasou:** Writing – original draft, Visualization. **Bahman Panahi:** Writing – original draft, Visualization, Formal analysis, Data curation, Conceptualization. **Ali Shahi:** Writing – original draft. **Yousef Nami:** Writing – review & editing, Writing – original draft.

## Declaration of competing interest

The authors declare there is not any conflict of interest.
